# “It is not something you like to hear, but it is something you have to know”. Community preferences for risk communication during pregnancy in the context of an emerging pathogen: a multicountry qualitative study with women in three ZIKV endemic countries who were pregnant during and following ZIKV

**DOI:** 10.1136/bmjgh-2025-020477

**Published:** 2026-06-19

**Authors:** María Consuelo Miranda Montoya, Claudia Milena Hormiga Sánchez, Ester Paiva Souto, Edna Acosta Pérez, Gustavo Matta, Juliana Silva Corrêa, Marcela Daza, Gabriela Gama, Camila Pimentel, Marcela Mercado-Reyes, Angelica María Amado Niño, Elena Marbán-Castro, Luz Marina Leegstra, Olivia Manders, Priscila Cardia Petra, Lauren Maxwell

**Affiliations:** 1 El Centro Internacional de Entrenamiento e Investigaciones Médicas (CIDEIM), Bucaramanga, Colombia; 2 Facultad de Salud, Departamento de Salud Pública, Universidad Industrial de Santander, Bucaramanga, Colombia; 3 Facultad de Ciencias de la Salud, Universidad Autónoma de Bucaramanga, Bucaramanga, Colombia; 4 Oswaldo Cruz Foundation (FIOCRUZ), Rio de Janeiro, Brazil; 5 University of Puerto Rico Medical Sciences Campus, Graduate School of Public Health, San Juan, Puerto Rico; 6 Hispanic Alliance for Clinical and Translational Research, San Juan, Puerto Rico; 7 Fundacao Oswaldo Cruz (FIOCRUZ), Policy and Administration in Health, Rio de Janeiro, Brazil; 8 Fundação Getulio Vargas, Rio de Janeiro, Brazil; 9 Instituto Nacional de Salud, Research Division, Bogota, Colombia; 10 Professor Joaquim Amorim Neto Research Institute (IPESQ), UNIFACISA University Center, Paraíba, Brazil; 11 Laboratory for studies on drugs, vulnerabilities and social markers (LED), Department of Public Health (NESC), Aggeu Magalhães Institute - Fiocruz Pernambuco, Recife, Brazil; 12 Dirección de Investigación en Salud Pública, Instituto Nacional de Salud, Bogota, Colombia; 13 ISGlobal, Hospital Clínic-Universitat de Barcelona, Barcelona, Spain; 14 Heidelberger Institut für Global Health, Universitätsklinikum Heidelberg, Heidelberg, Germany; 15 Rollins School of Public Health, Hubert Department of Global Health, Emory University, Atlanta, Georgia, USA; 16 Postgraduate Program in Bioethics, Applied Ethics and Collective Health (ENSP-FIOCRUZ); Interdisciplinary Centre for Public Health Emergencies (NIESP-CEE-FIOCRUZ), Oswaldo Cruz Foundation, Rio de Janeiro, Brazil

**Keywords:** Decision Making, Global Health, Health services research, Maternal health, Arboviruses

## Abstract

**Introduction:**

Community values and preferences should inform risk communication during pregnancy in the context of an epidemic of an emerging pathogen, given the high levels of uncertainty in the diagnosis and the probability of an adverse outcome.

**Methods:**

We conducted a multicountry qualitative study with 98 women from 7 sites in Brazil, Colombia and Puerto Rico to understand their preferences for learning about Zika virus test results amid high levels of uncertainty in diagnostic testing and related outcomes. We used thematic analysis with a combined deductive and inductive approach to analyse findings from the in-depth interviews.

**Findings:**

Across sites and time periods, women wanted to learn about their test results, the risk to their pregnancy or infant and the related uncertainty as early as possible. They wanted to separate risk communication from decision-making around the following steps to give them time to consider the test results, related risks and available services.

**Conclusions:**

Communication of the risk associated with an emerging pathogen suspected to affect pregnancy and developmental outcomes is a fraught issue. Public health authorities and healthcare providers should work closely together to understand families’ preferences for risk communication in the presence of uncertainty and develop a community-informed plan for risk communication as early as possible during an epidemic of an emerging infectious disease.

**WHAT IS ALREADY KNOWN ON THIS TOPIC**

- Risk communication during pregnancy regarding an emerging pathogen thought to affect fetal development must consider individual and collective values and preferences, as well as how to clearly express immediate and longer-term risks in the context of a high level of uncertainty.

**WHAT THIS STUDY ADDS**

- Understanding variations in women’s preferences related to risk communication, including the timing and context of communication in the presence of high levels of uncertainty, can help ensure that clinical risk prediction tools are informed by the sociocultural environment in which they are administered.

**HOW THIS STUDY MIGHT AFFECT RESEARCH, PRACTICE OR POLICY**

- Transparent, woman-centred risk communication between the health professional and the pregnant woman should be prioritised as a central element of prepregnancy care during the health system response to an emerging pathogen thought to affect pregnant women or fetal and infant development.
- How and by whom the news about the diagnosis of adverse findings is delivered is important for pregnant women and their families. Respectful, planned communication by a trusted provider can help pregnant women and their families understand complex topics and feel more confident in their decisions despite high levels of uncertainty.
- Ensuring pregnant women and their families’ needs are met should include special follow-up and psychosocial support for pregnant women with suspected Zika infection.

## Background

 The WHO interim guidance on Zika virus (ZIKV) communication was released in March 2016 when researchers were still questioning the link between ZIKV infection during pregnancy and adverse fetal or infant outcomes.^1^ In the intervening years, primary ZIKV infection during pregnancy has been causally linked to adverse fetal and infant outcomes, including congenital Zika syndrome (CZS), a spectrum of cognitive, speech and motor-related disabilities.^1^ While initially associated with congenital disabilities, ZIKV is now known to cause postnatal onset microcephaly and an array of additional cognitive or behavioural delays or disorders that are not observable in infants.^2^

Despite the high level of investment in ZIKV diagnostics and in studies of pregnant women exposed to ZIKV, there is a high degree of uncertainty, both in the accuracy of the ZIKV diagnostics and in the likelihood that a confirmed infection will be associated with adverse pregnancy or developmental outcomes.^3^ In 2016, WHO and the Pan-American Health Organization (PAHO) led the initiation of an international collaboration of ZIKV researchers to conduct an individual participant data meta-analysis (IPD-MA) to leverage all existing de-identified, subject-level data to estimate the absolute risk of adverse fetal, infant and child outcomes associated with ZIKV infection during pregnancy across cohort studies of pregnant women and their infants and children, the ZIKV IPD Consortium.^4^ The ZIKV IPD Consortium is developing and evaluating a clinical risk prediction model to identify high-risk pregnancies, inform individual women’s and family planning efforts and otherwise improve clinical and public health communication around the risk of ZIKV infection during pregnancy.

Risk communication in public health is the exchange of information, advice and opinions between healthcare providers or public health officials and the people facing the health concern, that is, the probability that a particular hazard will cause an effect. Risk communication, including its informational and communication components, must be contextualised by individual and collective values and needs.^5^ Risk communication is central to healthcare communication and patient autonomy^6^ and is a key pillar of outbreak response.^6^

When communicating risks, healthcare providers must consider the probability, level (ie, high or low), effect and dimensionality (ie, individual or population level)^7^ of the risk, as well as individual and community values.^5^ Risk communication is especially difficult in the context of emerging infectious diseases because of the high level of uncertainty regarding the probability of diagnosis, the probability of disease and the relationship between infection and disease.^8^ Healthcare providers and policymakers must ensure that the community at risk can make informed decisions to mitigate the risk of infection or related sequelae, even without confidence in the risks or in the accuracy of the diagnosis itself.

### Objective

Providing accurate, evidence-based information to women and families during an emerging infectious disease outbreak is complicated by the high degree of uncertainty in both diagnosis and risk. In another manuscript,^9^ we summarise study findings on what pregnant women were told during the ZIKV epidemic about their ZIKV test results and the risk of adverse fetal or infant outcomes. Findings from this study on what and how women of reproductive age in ZIKV-affected countries would like to receive diagnostic and outcome-related information will be used to contextualise the communication of findings from the predictive models developed by the global ZIKV IPD Consortium IPD-MA. The preferences of women of reproductive age from ZIKV-affected areas will be used to inform future health communication about the risk of ZIKV during pregnancy.

## Methods

### Design and study population

We conducted a cross-sectional, qualitative study with women of reproductive age in three of the countries most affected by the 2015–2017 ZIKV epidemic to understand women’s values and preferences for learning about test results and the probability of adverse fetal, infant or long-term outcomes during pregnancy in the presence of high levels of uncertainty. We included women pregnant during and after the ZIKV epidemic to understand whether values and preferences differed between these two groups. We conducted the study in seven locations: Campina Grande, Recife and Rio de Janeiro in Brazil; Bucaramanga, Neiva and Barranquilla in Colombia; and throughout Puerto Rico. The selection of sites for this study was based on several criteria, including the high incidence of ZIKA infection during the outbreak, the burden of ZIKV-related CZS, differences in access to contraception and legal abortion, and varying levels of gender equity, economic resources and religiosity. Interviews were conducted between April 2020 and July 2021, during the COVID-19 pandemic. Experiences caring for children with CZS during COVID-19 were addressed in the first section of the interview guide and will be reported in a subsequent manuscript. Site-level data collection periods are provided in online supplemental table S1.

### Study population

Participants were women aged 18 and above who resided in areas affected by the ZIKV 2015–2017 epidemic. Participants were grouped by (1) women who were pregnant during ZIKV and who had a child with at least one of the signs of CZS; (2) women who were pregnant during ZIKV and had a child without apparent signs of CZS and (3) women who were pregnant during the COVID-19 epidemic (July 2020–July 2021) rather than during the ZIKV epidemic. Participants who were pregnant during ZIKV were identified through health clinics, government services, social media and community-based organisations in Puerto Rico; through the ZIKV Cohorts participating in the IPD Consortium in Colombia and Campina Grande, Brazil; and through mothers’ organisations in Rio de Janeiro and Recife, Brazil. Participants who were pregnant during COVID-19 were identified through prenatal screening programmes. Further details on the source population, recruitment process, incentive provision and psychosocial support services during and after the interviews are included in online supplemental table S1.

### Data collection

The research team developed semistructured interview guides for each of the groups mentioned above. We reviewed the ZIKV and risk communication literature to develop the interview guides and spoke with infectious disease physicians about communicating about ZIKV infection during pregnancy. We divided the interview guides into five sections: (1) experiences with the COVID-19 pandemic, which was ongoing at the time of fieldwork; (2) ZIKV knowledge; (3) perceptions of risk; (4) the delivery of ZIKV results to pregnant women during the epidemic and (5) use of de-identified patient data for the WHO and PAHO-mediated IPD-MA. In this manuscript, we report on risk communication preferences. We pilot-tested the interview guides in Brazil with three women who would have been eligible to participate in the study to refine and clarify the guides. Following the pilot test, the interview guide was shortened, and some questions were restructured for improved clarity. We included the pilot test interviews in the overall analysis.

While we had initially planned to conduct in-person focus group discussions, we shifted to individual in-depth interviews due to the ongoing COVID-19 pandemic at the time of data collection. We conducted in-depth interviews via Zoom or WhatsApp, depending on participant preference, except for Campina Grande, Brazil, where in-person interviews were conducted after prenatal or wellness visits for currently pregnant women in keeping with local social distancing regulations to protect research participants during the COVID-19 pandemic. Interviews, which covered the five research objectives for the overall study, lasted between one and one and a half hours.

### Analysis

The in-depth interviews were conducted in Portuguese or Spanish and transcribed and analysed in their original languages. Deductive codes were derived from the interview guides, and inductive codes were developed through team discussions of each transcript. Thematic coding was done using Dedoose software (Sociocultural Research Consultants, Manhattan Beach, California, USA). The codebook is provided in online supplemental table S2. We ensured inter-coder agreement through weekly small-group and larger-team discussions to review the codes. We developed thick descriptions for a core set of codes related to our research questions. We alternated between data collection and analysis, allowing us to modify the interview guide during data collection to better capture emerging themes. Saturation was achieved within countries and separate groups (eg, pregnant during COVID-19 rather than the ZIKV epidemic) when no new participant types or issues emerged. Theory generation was based on comparative analyses of data collected from participants and their diverse profiles, contexts, and countries. Select supporting quotes are included in the text with their English-language translation. The original Spanish or Portuguese and the English language translation of quotes included in the text, and additional supporting quotes are included in online supplemental table S3. The study design and findings are reported in accordance with the Criteria for Reporting Qualitative Studies (COREQ; online supplemental table S4).

### Researcher characteristics and reflexivity

The research team comprised 17 members with diverse professional backgrounds, spanning community-based participatory research, sociology, counselling, qualitative and mixed-methods research, infectious disease diagnostics and epidemiology. The research team included investigators from the Universidad de Puerto Rico and the Carlos Albizu University in Puerto Rico, the Oswaldo Cruz Foundation (FIOCRUZ) and the Instituto de Pesquisa Professor Joaquim Amorim Neto (IPESQ) in Brazil, the Universidad Industrial de Santander, the Instituto Nacional de Salud, the Centro de Atención y Diagnóstico de Enfermedades e Infecciosas-CDI, Fundación INFOVIDA and the Universidad Autónoma de Bucaramanga in Colombia, many of whom led ZIKV-related cohorts of pregnant women and their infants and children. All team members were proficient in Spanish and Portuguese; transcripts were discussed and analysed in their original language. Except for four team members, the researchers were from the included countries and had established close connections with the study participants’ source communities. The BMJ Global Health Author Reflexivity Statement is included as online supplemental table S5.

## Results

Table 1 presents the study participant demographic information. 98 women from seven sites participated in the study (38 from Brazil, 39 from Puerto Rico and 21 from Colombia). A higher percentage of mothers in Brazil (55%, N=21) and Colombia (52%, N=11) reported having children with CZS compared with Puerto Rico (21%, N=8). The substantial proportion of mothers without CZS in Puerto Rico (41%, N=16) was related to the lower incidence of the syndrome in this region. Education levels varied across countries and sites. Puerto Rico had the highest percentage of participants with a graduate-level education (56%, N=22), followed by Brazil (47%, N=18) and Colombia (33%, N=7). Brazil had the most participants with only a primary/middle school education (18%), Colombia had none (0%) and Puerto Rico had 5%.

**Table 1:** Demographic characteristics (N=98)

Characteristic	Brazil (N=38)		Colombia (N=21)		Puerto Rico (N=39)		Total (N=98)	
Pregnant during ZIKV	N	(%)	N	(%)	N	(%)	N	(%)
Mothers of children with CZS	21	(55)	11	(52)	8	(21)	40	(41)
Mothers of children without CZS	10	(26)	7	(33)	16	(41)	33	(34)
Pregnant, currently pregnant during the COVID pandemic, not pregnant during the ZIKV epidemic	7	(18)	3	(14)	15	(38)	25	(26)
Age average		34		29		30		31
Level of education								
Graduate	18	(47)	7	(33)	22	(56)	47	(48)
Undergraduate	0	(0)	10	(47)	7	(18)	17	(17)
High school	13	(34)	4	(19)	8	(21)	25	(26)
Primary middle school	7	(18)	0	(0)	2	(5)	9	(9)
Marital status								
Married	18	(47)	7	(33)	16	(41)	41	(42)
Cohabitating	16	(42)	8	(38)	17	(44)	41	(42)
Single	4	(10)	5	(24)	6	(15)	15	(15)
Divorced	0	(0)	1	(5)	0	(0)	1	(1)

CZS, congenital Zika syndrome; ZIKV, Zika virus.

The average age reflects a relatively young maternal population, with Brazil having the highest average age (34 years). The percentages of married participants are similar across all three countries, with Brazil at 47% (N=18), Colombia at 33% (N=7) and Puerto Rico at 41% (N=16). The total percentage of married participants is 42% (N=41). There was greater variability in partnership status across sites. In Brazil, 10% (N=4); in Colombia, 24% (N=5); and in Puerto Rico, 15% (N=6) of respondents were single. In total, 15% of the sample were single (N=15).

### Communication of risk in the presence of uncertainty

Both pregnant women interviewed during the ZIKV epidemic (whose children were or were not affected) and those pregnant during the COVID-19 pandemic but not during ZIKV agreed on the ideal way for communicating risk related to an emerging pathogen that could adversely affect fetal development and infant and child outcomes. Participants felt that test results should be communicated with a clear description of their uncertainty and recommendations for repeat testing to address the possibility of false positives or negatives. Women preferred that results be delivered as early as possible in the pregnancy and that they be referred to a follow-up appointment to discuss questions and possible ways forward for addressing the challenge of a ZIKV-affected pregnancy, rather than having that discussion when they learnt about the test results (quotes 1–3, online supplemental table S3).

Interviewer: Should the mother be told about the effects of this infection on the pregnancy?

Participant: Yes, ma’am, I think so…it is good to know. It may be I would say that it would help a lot, at the moment, it would be scary, but it would be good to know.

Interviewer: And what else would you need to know?

Participant: What follow-up should she do during the pregnancy? There are people who are disappointed and decide not to have the baby. I have heard many like that, that they prefer to abort than to have a sick child, but there are other people who do accept it like that. (NOCZS008, Colombia, Pregnant during ZIKV, no adverse pregnancy outcome, 35 or over)

Interviewer: How should this risk be explained?

Participant: That’s complicated, isn’t it, because everything you are going to communicate to the person, you don't know the way you are going to act, don't you, but I think you have to say as much, it has to be as clear as possible, not to scare, to say that it’s going to happen, it’s like that, isn't it, not that it is, but that you are fully aware of the possibilities…It is not something you like to hear, but it is something you have to know, that there is a possibility, however much it hurts, whatever happens, ok, if there is, we will have to think, about how you are going to work, it is…this syndrome when it is born, what kind of things…If it does, physiotherapy, then you think from the other side, but you have an idea at least of what is coming in your life. I think you have to talk about it as frankly as possible. (BRID_GRJEP01, Brazil, Pregnant during COVID-19 pandemic, 35 or over)

Interviewer: What should health personnel say about the effects of Zika infection during pregnancy?

Participant: You should tell her what care she should take, if she should suddenly be in a safer place or suddenly if she could be infected, because suddenly the test has a margin of error, she can infect another person, suddenly where lives are not the optimal conditions, first tell her the conditions in which she should live, how she should feed herself, how she should take care of herself, because she can later present, explain to her the care in every detail. (PREG001, Colombia, Pregnant during COVID-19 pandemic, age 25-34)

### Communicating uncertain test results

Some participants emphasised the importance of explaining that a positive test result did not necessarily mean that the infant would be neurologically affected by Zika; maternal ZIKV infection is not synonymous with microcephaly. Several respondents associated this with their experience during COVID-19, in which a positive test result may not have indicated symptoms or other adverse effects (quote 4, online supplemental table S3).

In the event of a negative test result, participants from the three groups agreed that healthcare personnel should reinforce the utility of preventive measures to avoid infection, explain ZIKV symptoms and associated risks, and ideally, repeat the laboratory test later in pregnancy (quotes 5–8, online supplemental table S3). Respondents were aware of mosquito control measures but felt that providers could remind women about them.

Interviewer: And if the result indicates that there is no infection in the mother and we know that the tests are not always correct, what should the health professional tell her about this negative result?

Participant: Ah… you have to… you have to tell her that even if… it is negative she should take care of herself, that she should take care of herself with all possible care and that she should continue protecting herself, using repellent, that is… be careful with this issue of stagnant water, in short, in the traditional ways that we know the virus reproduces, right? (BRID_GPEBS01, Brazil, Pregnant during COVID-19 pandemic, 35 or over)

### Empathetic communication

All participants highlighted the need for empathetic communication of abnormal ultrasound results by a provider known to the patient, given the emotional impact of learning about ultrasound-detectable alterations.

Interviewer: What should health personnel tell the pregnant woman about her test? And how should they tell her?

Participant: Well, first, empathetic because they throw the soup out there as if this is a party and as if you don’t have feelings because, believe me, it’s happened to me several times. This one, at least in terms of Zika, I made a connection with the nurse, and she’s a sweetheart. She’s very empathetic. And, well, I was fortunate that she [told Interviewer], “Look, mom, sit down.” I mean, they told me calmly, but that they are very empathetic is the most important thing. (ID306, Puerto Rico, Mother of a child with CZS, age 25-34)

Participants identified the medical professional who carries out the prenatal check-up or follow-up, who already knows the pregnant woman’s medical history, as best positioned to inform the pregnant woman about the possibility of adverse fetal, infant or child outcomes. Participants asked that the test result and the possibility of an adverse outcome be delivered by an empathetic healthcare worker or support personnel (eg, a psychologist or social worker) rather than by a doctor or ultrasound technician who had not been trained in communicating sensitive results during pregnancy.

Well, the doctor has to be empathetic with the pregnant woman. Well, because it is not a simple situation to know the possible risks of a baby that is developing and to confront any motor or intellectual development problems that the baby may have, that may have special needs. And then to prepare the person emotionally for any complicated situation that may arise in the eventuality of a miscarriage or malformation of the baby. So it would be a lot of empathy and with much, much closer follow-up. (ID100, Puerto Rico, Pregnant during COVID-19 pandemic, 35 or over)

Participants emphasised that this professional should listen to the woman’s concerns and discuss the decision-making process and options with her without pushing any particular option, including abortion (quotes 8–9, online supplemental table S3). Participants from Brazil suggested that a neurologist communicate any adverse ultrasound findings with the woman to facilitate an informed discussion of the effect of congenital ZIKV exposure on infant and child development, while other participants felt that a psychologist or social worker should communicate the results.

I think the first step would be to see a psychologist within the unit itself or some social worker who can give you more support because we cannot leave the exam, the result in the patient’s lap, and talk. Let’s be honest. The pump is yours. You have to come with all the structure, right? The psychology and the assistance to be able to support this family, right? (BRID_SRJEP01, Brazil, Mother of a child with CZS, 35 or over)

Across sites, respondents felt that the healthcare team should carefully plan how to communicate adverse results rather than deliver them spontaneously on identification. Respondents preferred that communication about adverse outcomes be face-to-face, calm, empathetic and respectful. When communicating the potential adverse outcomes, respondents asked that healthcare providers or other support staff offer alternative ways to cope with them without judging or pitying the woman. Respondents requested that the information be delivered privately and that the woman be asked to bring someone with her to discuss the results (quotes 12–14, online supplemental table S3).

### Providing information about resources for women with ZIKV-affected pregnancies

Several of the interviewees from Puerto Rico stated that, in addition to the risks, the health system should provide clear, detailed information about the state support that would be received if the infant were affected by ZIKV, the support that would be received from the health system, and mechanisms for receiving social support from other mothers confronting the same challenges (quote 15, online supplemental table S3).

Interviewer: What else do you think a pregnant woman should know?

Participant: That there are more treatments for the child, that she will need more therapy and that the health system is prepared to have that baby when it arrives. That she will not be alone in this process.

Interviewer: Is there anything else the pregnant woman should be told?

Participant: That she will be taken care of, that there is a group of mothers who are going through the same risk, and that the mother will be well cared for… that she should feel hopeful that she is not the only one. It is not the same walking through a swamp alone as walking with three in front and two behind because you are accompanied. That makes the panorama different. (ID300, Puerto Rico, Mother of a child with CZS, 35 or over)

### Community preferences for how and when to inform a pregnant woman about the effect of ZIKV on her infant

The interviewees who were informed about the neurological impairment of their child at an advanced stage of pregnancy asked to be informed of any suspected alterations earlier in pregnancy, even if the findings could not be communicated with certainty. They felt that being informed early, even if findings were not certain, would allow the mother to prepare herself emotionally and ensure that she could gather information on how to care for a CZS-affected infant. Participants cited religious faith, information on resources to understand their child’s developmental trajectory, how to care for their child, and how to support their child’s development and publicly available support services for their CZS-affected infant, as ways that pregnant women would use to prepare themselves after learning that their infant had been exposed to ZIKV or having received evidence of fetal abnormalities. When given the option of receiving uncertain information on potential exposure to or effects of maternal ZIKV exposure early in pregnancy to receiving a more certain diagnosis of CZS late in pregnancy, participants across respondent groups and geographies preferred to learn about the less certain exposure earlier in pregnancy rather than learn about a more certain exposure or outcome later in pregnancy to give them time to prepare emotionally and practically for a ZIKV-affected infant (quotes 16–18, online supplemental table S3). Participants also wanted to be told about the possibility of adverse developmental outcomes that could manifest after birth, even given a high level of uncertainty.

## Discussion

We conducted a cross-sectional qualitative study in three ZIKV-endemic countries to understand how women of reproductive age would like to be informed about the risk of an emerging pathogen thought to adversely affect pregnancy when there is a high degree of uncertainty in the diagnosis of infection and the probability of an adverse outcome. We included women from seven sites in three countries to capture perspectives from women in regions with different levels of religiosity, access to safe abortion and other factors that could affect pregnancy care-related decision-making or risk communication preferences. While we found that women in different countries had markedly different experiences regarding how test results and adverse outcomes were communicated during ZIKV,^9^ we did not find meaningful differences in how women wanted to learn about test results or related outcomes during pregnancy amid high levels of uncertainty.

A number of participants reported that learning about CZS during an ultrasound or being presented with abortion as the only option led to high levels of stress and distrust of the health system. These findings are consistent with a UK study showing that risk communication that challenges pregnant women’s autonomy can reduce trust in healthcare providers.^10^ Participants preferred to learn about potential fetal abnormalities earlier with a higher degree of uncertainty than to receive more certain findings later, reporting that early communication would facilitate emotional and logistical preparation for caring for an infant with CZS. This aligns with quantitative and qualitative studies across Latin America and the Caribbean that recommended enhancing public health messaging about ZIKV during the epidemic.^11^,^17^ Given the importance of ultrasound in ZIKV risk communication, the communication skills of ultrasound professionals should be strengthened so that, in coordination with the treating clinician, they can provide clear, understandable information in an empathetic, personable manner. In keeping with prior research,^18^ participants reported that respectful, empathetic communication could help women and their families feel better prepared to cope with an adverse result.

Participants’ desire to establish meaningful, informed dialogue with the professional who discusses the test or outcome-related results, the desire to be accompanied by a family member or friend when learning about results, and the need for emotional support after learning about adverse findings have been reported in another ZIKV-related study.^18^ Such dialogue requires health professionals to develop communication skills that enable horizontal relationships with patients, guided by respect for cultural and religious beliefs, views on disability, pregnancy termination, and other relevant topics.

**Figure 1:**
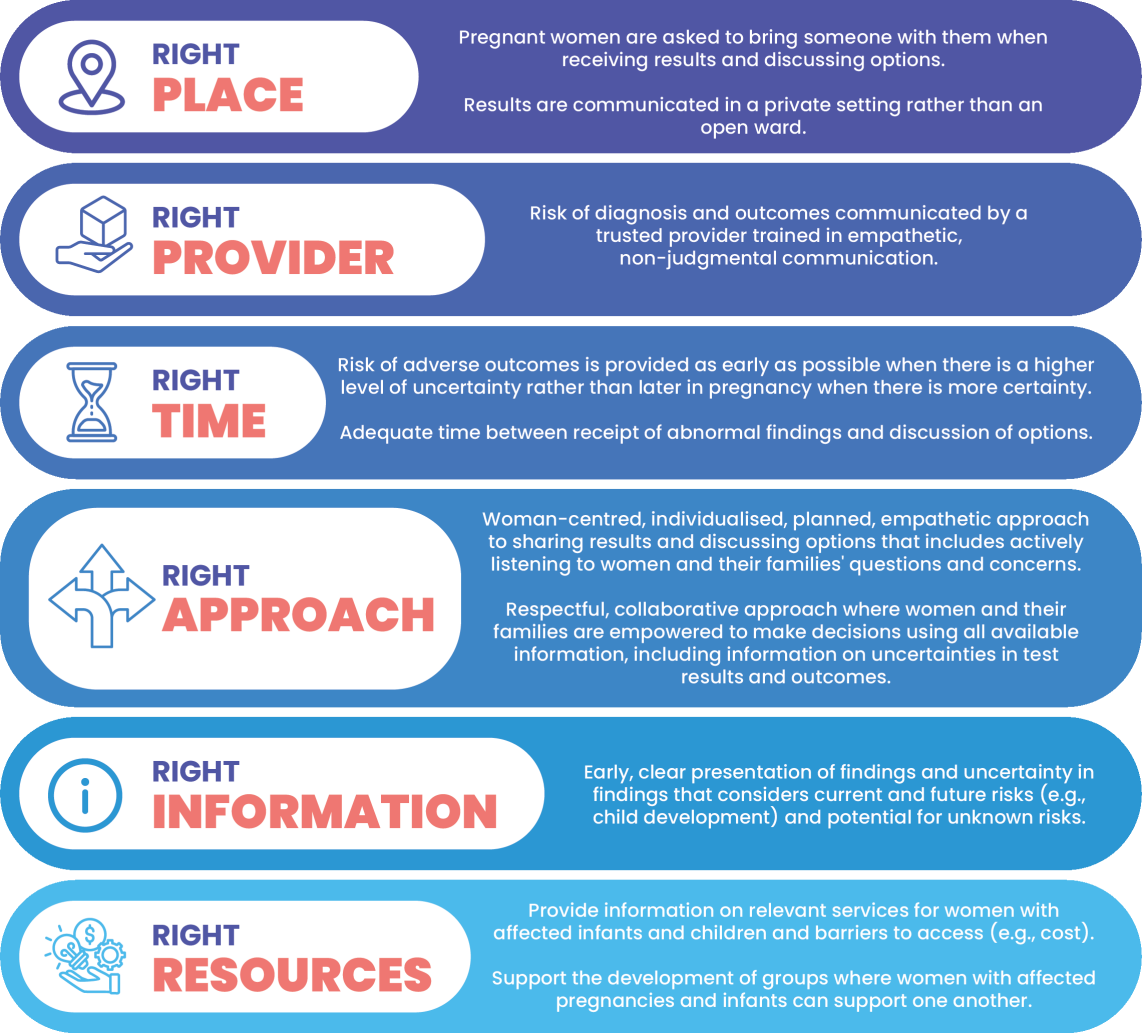
Participant preferences for patient-centred, values-based risk communication during an emerging pathogen affecting fetal development.

Figure 1 summarises participant preferences for a woman-centred, values-based approach to risk communication during an emerging pathogen affecting fetal development. Rather than limiting information to the risk of a congenital abnormality, participants wanted risk communication to include information about services available for their ZIKV-affected infant (eg, state aid), support networks and practical guidance on addressing their child’s special needs.

Best practices for risk communication before and during pregnancy, and ethical issues related to communicating screening and diagnostic test results during pregnancy, come almost exclusively from the genetic testing literature, where probabilities are well established, and test inaccuracy can be clearly communicated.^19^ We conducted a systematic search but found only seven articles addressing infectious disease-related risk communication during pregnancy from the perspective of pregnant women. Participants in these studies expressed having received little information about ZIKV and the risk to their pregnancy, which increased anxiety.^20^,^22^

Given the high level of uncertainty related to both whether a pregnant woman has an incident ZIKV infection and whether, given infection during pregnancy, her fetus, infant, or child will experience an adverse ZIKV-related outcome, individual providers, laboratory staff and ministries of health did not have a clear path forward for communicating either ZIKV-related assay results or ZIKV-related risks to pregnant women and girls who were tested for ZIKV. When the validity of the available tests is not established, the health system or individual providers may choose not to communicate diagnostic test results to pregnant women, as occurred in some settings during the ZIKV epidemic.^23^ Many of the countries hardest hit by the 2015–2017 ZIKV epidemic had very restrictive access to pregnancy termination services,^24^ which, coupled with the high levels of uncertainty in the ZIKV assays, may have deterred providers and health systems from sharing assay results with pregnant women. This contrasts with WHO guidance recommending that risk communication in healthcare settings be clear, transparent, and empathetic.^25^

Effective risk communication during an epidemic requires understanding the audience, building trust, providing timely and accurate information, and addressing misinformation.^25^ Communication should be culturally sensitive and consider the diversity of patient values and preferences, particularly around pregnancy termination. Pregnant women and their families should be central to the discussion on how to best communicate the probabilities and uncertainty in diagnostic tests and adverse outcomes associated with infection in the context of an emerging pathogen. Effective risk communication must engage communities, empower individuals to make informed decisions and foster an environment where feedback is welcomed and acted on.

No risks were known with certainty, and certain outcomes were not known at all to clinicians when they had to communicate diagnostic or fetal findings to pregnant women during the ZIKV epidemic, including the postnatal onset of microcephaly related to viral replication in the fetal brain. How to help pregnant women retain autonomy and trust in the health system during risk communication amid such high levels of uncertainty remains largely unexplored in the literature and warrants consideration in future studies.

### Strengths and limitations

This study’s key strength is that it directly engaged the community members most affected by the ZIKV outbreak, and the future target audience for clinical risk prediction tools, ensuring that women’s preferences informed our analysis. The diversity of the sample enabled cross-country and cross-site comparisons, and the study’s design incorporated four of the five WHO-recommended communication pathways for ZIKV-related risks: translational communication, stakeholder coordination, community engagement and dynamic listening.^26^

Limitations include the delay between the ZIKV epidemic and fieldwork, due to ethics review processes that coincided with the COVID-19 pandemic, which may have affected recall of risk communication preferences. We were unable to conduct focus group discussions as planned and shifted to online in-depth interviews, which may have reduced data richness or limited participation by women with heavy caregiving responsibilities, though remote qualitative methods can yield meaningful engagement.^27^ We also did not explore preferences regarding how to communicate test sensitivity and specificity, as ZIKV results were not reported with probabilities in study settings during the epidemic.^2^ Future research should develop guidance on communicating test properties and probabilities in rapidly evolving evidence contexts.

### Policy implications

Given prior research indicating that risk is best communicated through visual and written aids rather than purely oral communication,^28 29^ future research could investigate how to convey uncertain results (eg, inconclusive tests) in the context of an emerging pathogen. Mechanisms for incorporating patient feedback into risk communication during public health emergencies have not been well developed. Pregnancy termination and caring for a severely disabled infant in a resource-limited environment are important concerns that need to be discussed in keeping with local values, preferences, legal frameworks and health and social support system capabilities. Future work is needed to explore how to rapidly engage the community in creating decision aids and to ensure that pregnant women have sufficient information and resources to guide decision-making. The way the diagnosis is provided should be planned to support and clarify any doubts that may arise about the pregnancy and the impact of the infection on infant and child outcomes,^18^ along with an honest assessment of the provider’s uncertainty.

Risk communication must be tailored to the specific religious, cultural and legal context.^26^ We did not find important differences in how women wanted to learn about their ZIKV-related risks or the level of uncertainty in that communication, despite the differences in access to abortion and levels of religiosity across the sites and countries included in the study. Our sample was relatively limited in terms of minority status and education level. Future research should continue to explore potential sources of heterogeneity (eg, maternal age, parity, education, religiosity and minority status) in preferences for risk communication strategies in the context of an emerging pathogen.

## Conclusions

Risk communication in the context of an emerging pathogen that adversely affects fetal development should be grounded in community preferences for learning about diagnostic test uncertainty and the associated probability of adverse outcomes. Findings from this study can inform risk communication for infectious disease exposures affecting pregnant women when no gold-standard diagnostic exists and should be integrated into the development of clinical risk prediction tools as a central component of ethical, person-centred care.

## Supplementary material

10.1136/bmjgh-2025-020477 online supplemental table 1

10.1136/bmjgh-2025-020477 online supplemental table 2

10.1136/bmjgh-2025-020477 online supplemental table 3

10.1136/bmjgh-2025-020477 online supplemental table 4

10.1136/bmjgh-2025-020477 online supplemental file 1

## Data Availability

Data are available on reasonable request.
