# Supplementary material for: “It is not something you like to hear, but it is something you have to know”. Community preferences for risk communication during pregnancy in the context of an emerging pathogen: a multicountry qualitative study with women in three ZIKV endemic countries who were pregnant during and following ZIKV
Source: BMJ Glob Health. 2026 Jun 19;11(6):e020477. doi: 10.1136/bmjgh-2025-020477 (PMC13289160; doi:10.1136/bmjgh-2025-020477)
Supplement: online supplemental table 2 [file bmjgh-11-6-s002.docx]

Supplementary Table S2. Codebook

| **Code** | **Description** |
| --- | --- |
| **Actual Communication of Results** | Use for any discussion about how women learned about their ZIKV test results not mentioned in the child codes. This includes positive and/or negative tests or an ultrasound. |
| **Actual Communication of Test Results** | Use for any description of how women were told about their ZIKA test result (or the lack of them). Use for a description of the experience. E.g., who told them, where they were, how they were told, and who was with them.   Also use for description of any discussion with the provider about the possibility (chance) that ZIKA could affect the pregnancy and/or fetus/baby. This includes the chance that ZIKA might affect the pregnancy, fetus or infant/child. Also, use to mention that this discussion DID NOT take place or this information was not communicated to the participant. |
| **Actual Negative test / ultrasound result / Resultados reales test negativo /** | Use for the description of the doctor's certainty that the test result was negative. Include a description of WHY the doctor said they thought it was negative, and discuss their certainty/incertainty of the diagnosis. Include mention of the possibility of negative being positive.   Double code with Uncertainty. |
| **Actual Positive test / ultrasound result / Resultados reales test positivo /** | Use for description about the doctor's certainty that the test result was positive. Include for description of WHY the doctor thought it was positive, and description of what a positive test would mean. Include possible impact to the pregnancy, fetus, infant/child. |
| **Provider Certainty/Uncertainty** | Participant's description of healthcare provider's certainty or uncertainty about the accuracy of a positive or negative test result. |
| **Challenges** | Use for any discussion about the CHALLENGES of having a baby or child affected by ZIKV/with CZS. |
| **CZS Description** | Use for description of HOW Zika affected their infant/child. |
| **Emotions, Feelings, Uncertainty** | Use for indication of how women feel about their situation, their fetus/infant/child's situation. Use for any mention of their feelings in the past or present. This includes the mention of uncertainty about possible outcomes or information. |
| **Ethics** | Use for mention of things related to ethics, including use of data and consent, ways women were informed about their test results, what women were told about their test results, voluntary participation in studies, if they were told that their data would be used, etc. |
| **Faith** | Use for mention of spiritual faith. |
| **Good Quotes** | Use for quotes that can be used for examples in the manuscripts or for providing guidance to providers. |
| **Memory / Passage of Time** | Use for mention that a lot of time has passed since Zika, they don't remember, or it is hard/difficult to remember. |
| **Recommendations** | Use for mention of recommendations made by the provider during or after the pregnancy. |
| **SES** | Use for discussion about aspects of their SES that might impact their understanding of or ability to prevent Zika. Also, use to see how SES impacts their ability to care for a CZS-affected child. |
| **Stigma** | Use for mention of Zika-related stigma, or fear of stigma related to ZIKV or having a CZS-affected child. |
| **Ideal Communication of Results** | Use for any discussion about the ideal way to communicate results. |
| **First heard about Zika** | Use for discussion of when the participant first heard or learned about Zika. This includes WHEN they hear about it.  When was the first time you heard about Zika? |
| **Zika Experience** | Use for any discussion about Zika (e.g., their symptoms, how they felt, what they did) that is not included in any of the codes. |
| **Additional comments** | Use for final additional comments that participants share.   Double code with any other relevant codes. |
| **Comments for Researchers** | Use for the one thing participants would like to tell researchers about ZIKA.   Double code with any other relevant codes. |
| **Zika Knowledge** | Use this code for any discussion of participant knowledge about Zika that is not related to one of the codes below. |
| **Zika Perceptions of Risk** | Use for any discussion about risk or perception of risk of Zika that is not included in the sub/child codes below. |
| **Risk to Pregnancy/Fetus (General)** | Use for discussion of what they heard about Zika infection in pregnant women from any source, and what could happen to the pregnancy and/or fetus. Also use for comparison to other diseases. |
| **Defining ZIKV risk through comparison with other illnesses** | Any social/cultural/biomedical description of illness during pregnancy that could have negative consequences on reproductive health, i.e. STORCH infections (Syphilis, Toxoplasma, Rubella, CMV, HIV, etc.); arboviral diseases (DENV, CHIKV, etc.), and others (genetic disorders, etc.). |
| **Risk to Infant/Child** | Use for discussion of what they heard about Zika infection in pregnant women from any source, and what could happen to the development of their child. |
| **Risk to Own pregnancy** | Use for discussion of how women thought their OWN pregnancy could be affected by ZIKA, based on what they had heard about what could happen if a pregnant woman became infected with Zika and what that could do to their pregnancy/fetus/child's development later on. |
| **Therapeutic Itinerary** | Use this for discussion about the pathways (journey) taken within the healthcare system to secure information and obtain care, resources, and treatment.   The term “therapeutic itinerary” (TI) refers to the search for treatment and seeks to describe and analyse individual and socio-cultural practices, in terms of the paths taken by individuals to solve their health problems, , including the logic that drives this pursuit, which is woven in multiple formal and informal networks of support to which a person belongs |
