# Supplementary material for: “It is not something you like to hear, but it is something you have to know”. Community preferences for risk communication during pregnancy in the context of an emerging pathogen: a multicountry qualitative study with women in three ZIKV endemic countries who were pregnant during and following ZIKV
Source: BMJ Glob Health. 2026 Jun 19;11(6):e020477. doi: 10.1136/bmjgh-2025-020477 (PMC13289160; doi:10.1136/bmjgh-2025-020477)
Supplement: online supplemental file 1 [file bmjgh-11-6-s005.docx]

### Supplementary Table S5. BMJ Global Health Author Reflexivity Statement

Adapted from Morton, B., Vercueil, A., Masekela, R., Heinz, E., Reimer, L., Saleh, S., Kalinga, C., Seekles, M., Biccard, B., Chakaya, J., Abimbola, S., Obasi, A. and Oriyo, N. (2022), Consensus statement on measures to promote equitable authorship in the publication of research from international partnerships. Anaesthesia, 77: 264-276. <https://doi.org/10.1111/anae.15597>

| **Study conceptualisation** | |
| --- | --- |
| 1. How does this study address local research and policy priorities? | This study was designed to address a critical gap identified during the 2015–2017 ZIKV epidemic: the absence of community-informed, culturally appropriate guidance for communicating diagnostic test results and adverse outcome probabilities to pregnant women under conditions of high uncertainty. Study sites were selected based on their direct experience of the ZIKV outbreak and the burden of congenital Zika syndrome (CZS). Findings are intended to inform risk communication guidance for Ministries of Health and healthcare providers in Brazil, Colombia, and Puerto Rico, and to contextualise output from the ZIKV IPD Consortium’s clinical risk prediction models. |
| 1. How were local researchers involved in study design? | The 17-member research team was predominantly composed of researchers from Brazil, Colombia, and Puerto Rico. Local investigators from the Universidad Industrial de Santander, Instituto Nacional de Salud, Fundación INFOVIDA, Universidad Autónoma de Bucaramanga, Universidad de Puerto Rico, Carlos Albizu University, and multiple FIOCRUZ institutes were involved in study conceptualisation, interview guide development, piloting, data collection, and analysis. Many had led ZIKV-related cohorts of pregnant women and their infants, providing direct expertise in the local research and policy context |
| **Research management** | |
| 1. How has funding been used to support the local research team(s)? | The study conceptualisation and field data collection was funded by a DFID/Wellcome Trust grant (HRP, grant 216002/Z/19/Z), with additional support from the Instituto Nacional de Salud, INFOVIDA, and the Centro de Atención y Diagnóstico de Enfermedades Infecciosas in Colombia, and the Hispanic Alliance for Clinical and Translational Research (NIH/NIGMS, grant U54GM133807) in Puerto Rico. These sources supported researchers and data collection staff at local institutions across all three countries and did not provide any funding for resesarchers other than local teams. |
| **Data acquisition and analysis** | |
| 1. How are research staff who conducted data collection acknowledged? | Atisha A. Gómez-Reyes and Vivian Torres (University of Puerto Rico) are explicitly acknowledged for their support with data collection. Other researchers who collected data also contributed to the analysis and writing and are included as co-authors. |
| 1. How have members of the research partnership been provided with access to study data? | All authors had access to the study data and were engaged in the analysis of these data. |
| 1. How were data used to develop analytical skills within the partnership? | Most authors had not conducted a qualitative analysis by the time we began this study. During this work, we worked closely with all authors to introduce the qualitative analysis software and to co-develop the thematic analysis.  The analysis involved weekly small-group and larger-team discussions to review codes and ensure inter-coder agreement, conducted across the full partnership. Thematic coding was done collaboratively using Dedoose software, with deductive codes derived from the interview guides and inductive codes developed through team discussion. Transcripts were analysed in their original languages (Portuguese and Spanish), and all meetings were conducted in both Spanish and Portuguese.  Capacity building in qualitative analysis from design to publication was an explicit goal of this project. |
| **Data interpretation** | |
| 1. How have research partners collaborated in interpreting study data? | Data interpretation involved iterative team discussions across the full partnership. The team alternated between data collection and analysis, modifying the interview guide during data collection to capture emerging themes. Saturation was assessed separately within countries and participant groups. Comparative analyses across diverse profiles, contexts, and countries, involving all authors, formed the basis of theory generation. |
| **Drafting and revising for intellectual content** | |
| 1. How were research partners supported to develop writing skills? | Several co-authors contributed to writing the manuscript. We did not envision or support academic writing workshops but rather focused on building capacity in the design, conduct, and analysis of qualitative research with vulnerable populations. |
| 1. How will research products be shared to address local needs? | Findings are intended to inform risk communication guidance for public health authorities and healthcare providers in the affected countries. Open access publication is supported by Heidelberg University Library, ensuring findings are freely available to policymakers and practitioners in Brazil, Colombia, and Puerto Rico  Our hope is that the findings will be disseminated through national partners with an explicit mandate to serve the populations included in this study, i.e., FIOCRUZ and the Colombian Ministry of Health, and that study authors will disseminate them at local meetings, conferences, and in global fora. |
| **Authorship** | |
| 1. How is the leadership, contribution and ownership of this work by LMIC researchers recognised within the authorship? | The first author is an infectious disease researcher based in Cali, Colombia. With the exception of 4 researchers, all researchers are based in Colombia, Brazil, and Puerto Rico, where the research was conducted. The distribution and order of authors reflect the project's local leadership from inception to dissemination. |
| 1. How have early career researchers across the partnership been included within the authorship team? | This study involved several early-career researchers who had been trained in qualitative analysis and supported the capacity-strengthening work, specifically Elena Marbán Castro and Luz Marina Leegstra. One early-career researcher at FIOCRUZ was involved in the study design: Juliana Correa. |
| 1. How has gender balance been addressed within the authorship? | The authorship team is predominantly female, consistent with the study’s focus on women’s perspectives and the leadership roles held by women researchers across the partnership institutions. |
| **Training** | |
| 1. How has the project contributed to training of LMIC researchers? | The collaborative analytical approach, including weekly coding discussions, inter-coder agreement processes, and iterative analysis, contributed to the development of qualitative research capacity among team members in Brazil, Colombia, and Puerto Rico. The interview guide pilot-testing process, conducted in Brazil with three eligible women, also provided practical training in qualitative instrument development. |
| **Infrastructure** | |
| 1. How has the project contributed to improvements in local infrastructure? | Our hope is that the study findings support a paradigm shift from risk communication with vulnerable populations under uncertainty in a public health emergency as a hierarchical process in which uncertain results are withheld, to a women-centred, respectful approach in which the values and preferences of the local population are foregrounded. |
| **Governance** | |
| 1. What safeguarding procedures were used to protect local study participants and researchers? | The study protocol was reviewed and approved by multiple independent ethics committees prior to data collection: the WHO ERC, Emory University IRB, Brazil’s CONEP, CEIB-CDI and CEMIN-INS in Colombia, and the BRANY SBER IRB in Puerto Rico. Informed consent was obtained from all participants for participation and publication of findings. In-person interviews in Campina Grande were conducted in accordance with local COVID-19 social distancing regulations. Psychosocial support services were made available during and after interviews (Appendix Table S1). |
